# Blood Pressure Variability during Angiography in Patients with Ischemic Stroke and Intracranial Artery Stenosis

**DOI:** 10.1155/2020/6214581

**Published:** 2020-09-01

**Authors:** Hui Pan, Rong Zhao, Feng-Di Liu, Yi-Lan Wu, Ge-Fei Li, Yan-Hui Shi, Yi-Sheng Liu, Ying Zhao, Mei-Ting Zhuang, Tian-Yu Hou, Qi-Ting Zhang, Qian Yao, Yuan Qiao, Rui-Jue Huang, Li-Na Chen, Yi-Min Zhu, Liang Shu, Jing-Jing Su, Jie Fang, Xue-Mei Tang, Shuo Wang, Guo-Hong Cui, David Z. Wang, Jian-Ren Liu

**Affiliations:** ^1^Department of Neurology, Shanghai Ninth People's Hospital, Shanghai Jiao Tong University School of Medicine, Shanghai 200011, China; ^2^Clinical Research Center, Shanghai Jiao Tong University School of Medicine, Shanghai 200011, China; ^3^Department of Epidemiology and Biostatistics, Zhejiang University School of Medicine, Hangzhou, China; ^4^OSF/INI Comprehensive Stroke Center, Department of Neurology, University of Illinois College of Medicine Peoria, Peoria, IL, USA

## Abstract

Our aim was to investigate factors predicting blood pressure (BP) variability during diagnostic cerebral angiography and associations between BP variability and clinical outcomes in patients with acute and subacute ischemic stroke and intracranial artery stenosis. 114 patients with ischemic stroke and intracranial artery stenosis (stenosis rate >50%) were recruited. Patients who underwent cerebral angiography within 3 days and 3–14 days of disease onset are referred to be Group A and Group S, respectively. BP variability in Group A was defined as the coefficient of variance (CV) of BP. Univariate and multivariate regression analyses were used to identify predictors of CV of BP and associations between CV of BP and clinical outcomes at discharge. In Group A patients, advanced age was associated with increased CV of SBP and diastolic blood pressure (DBP), and antihypertensive use was associated with lower CV of SBP. Male was associated with lower CV of DBP. In Group S, higher CV of SBP was associated with hypertension and antihypertensive use. Males had lower CV of SBP than females. The calcium channel blocker was associated with lower CV of DBP. Higher scores of the Stroke Scale at admission were significantly associated with poor clinical outcomes for both groups, while BP variability was not. Factors associated with BP variability are significantly different between stroke patients undergoing angiography within 3 days vs. 3–14 days after disease onset. BP variability is not significantly associated with clinical outcomes at discharge.

## 1. Introduction

Blood pressure (BP) variability and instability, including visit-to-visit BP variability, night-time BP variability, nocturnal hypertension, and stress-related hypertension (white-coat hypertension), are reported to be associated with adverse cardiovascular events and poor clinical outcomes [1–6]. BP variability during the acute stroke phase is also associated with poor clinical outcomes [7–9]. Kang et al. [8] showed that the average BP in the subacute stage of ischemic stroke influenced functional outcomes at three months after stroke onset and BP variability was suggested to be a potential independent predictor of clinical outcomes. Significant oscillations of BP occur both short- and long-term, and BP variability is a noted risk factor for stroke, independent of mean systolic blood pressure (SBP) [4]. Higher awake BP variability in Chinese patients with acute atherosclerotic stroke was also shown to be highly associated with large artery atherosclerosis [10]. Additionally, increased long-term, midterm, and short-term variability in SBP is significantly associated with mortality independent of mean BP [11]. BP variability is more detrimental when an internal carotid artery stenosis associate with ipsilateral stroke is linked to hypoperfusion [7]. In patients with lower BP and proximal vessel occlusion, increased BP variability is associated with worse outcome after ischemic stroke [12].

Diagnosis and treatment of patients with acute stroke have become increasingly reliant on interventional procedures. The standard of care for acute ischemic stroke includes intravenous thrombolysis and endovascular thrombectomy, both of which have resulted in good outcomes when treatment was initiated in a timely manner [13]. We have observed in clinical practice that stroke patients who undergo conventional cerebral angiography under local anesthesia frequently experience BP elevation. However, this kind of BP variability during catheter angiography has not been well studied in stroke patients, especially in stroke patients with intracranial artery stenosis. The question is whether this type of BP variability before and during the angiography procedure may influence clinical outcomes in this kind of patients.

In a cohort of acute ischemic stroke, patients with ipsilateral internal carotid artery (ICA) occlusion BP variability, assessed in the acute phase, were associated with poor clinical outcome. These preliminary exploratory findings are worthy of further study to be conducted to confirm or confute the role of BP variability in predicting stroke outcome. In order to obtain more comprehensive information, it would also be appropriate to consider the possibility of acquiring data related to the pathophysiology of stroke and to cerebral hemodynamic changes [7]. Therefore, acute stoke and intracranial stenosis might also be the cause of BP variability In this study, our aim was to explore factors predicting BP variability during diagnostic cerebral angiography and to evaluate associations between BP variability and clinical outcomes in patients with acute and subacute ischemic stroke and intracranial artery stenosis.

## 2. Patients and Methods

### 2.1. Patients

This prospective observational study recruited a total of 114 patients with acute and subacute ischemic stroke and intracranial artery stenosis (stenosis rate >50%) who received catheter cerebral angiography within two weeks of onset between March 2012 and November 2015. Patients were divided into two groups on the basis of the timing of cerebral angiography in relation to disease onset, as follows: patients who underwent cerebral angiography within 3 days of disease onset (Group A) and patients who underwent cerebral angiography 3–14 days after disease onset (Group S). Indications for angiogram included acute thrombectomy, further confirming diagnosis of intracranial artery stenosis. Cerebral angiography was performed under local anesthesia and with electrocardiographic monitoring. Patients who received general anesthesia during the procedure were excluded.

## 3. Methods

### 3.1. BP Monitoring

BP was recorded (1) upon admission, (2) immediately after entering the room in which the procedure would be performed (angio-room), (3) immediately before the procedure, and (4) every 15 min after the start of the procedure, using an upper arm cuff sphygmomanometer. Variability (coefficient of variance, CV) of BP during the procedure was calculated using the formula: variability of BP during procedure = (standard deviation/average) *∗* 100%.

### 3.2. Cerebral Angiography Procedure

Patients were placed in a supine position and received focal anesthesia (1% lidocaine) in the groin. The right femoral artery was punctured (left femoral artery in some exceptions). Whole cerebral angiography was performed with the 5F catheter using an isotonic nonionic iodine contrast agent. The contrast was diluted with normal saline at a ratio of 2 : 1 in order to reduce the stimulation to the blood vessel and the nervous system. During the procedure, heparin (1000 units of heparin in 500 ml of normal saline) was infused via a Y valve through the catheter. After angiography, an endovascular suture device (Perclose Proglide vascular stapler, Abbott Vascular, Abbott Park, IL, USA) was used for femoral artery wound closure. The wound was then compressed with an elastic bandage for further hemostasis.

### 3.3. Statistical Analysis

Demographics and clinical characteristics are presented as mean ± standard deviation (SD) or median (interquartile range, IQR) for numerical variables and frequency (%) for categorical variables. Simple linear regression analysis was performed to identify the effectors (including demographics and clinical characteristics) of coefficient of variation (CV) of preoperative SBP and diastolic blood pressure (DBP). Variables with significance level of *p* < 0.05 in the univariable model were then used to perform multivariable model analysis. For clinical outcome at discharge, results were represented as odds ratio (OR) with corresponding 95% confidence intervals (95% CI) for logistic regression analysis; for CV of preoperative SBP and DBP, results were represented as *β* (regression coefficient) with corresponding 95% CI for linear model analysis. When only one factor with *p* < 0.05 was found for an outcome in univariate analysis, multivariable analysis was not performed. All statistical assessments were two-tailed, and *p* values <0.05 were considered statistically significant. Statistical analyses were performed using SAS® 9.4 (Windows NT version, SAS Institute, Inc., Cary, NC, USA).

## 4. Results

### 4.1. Patients' Demographic and Clinical Characteristics

The present study recruited a total of 114 patients, including 33 patients in Group A who underwent cerebral angiography within 3 days of disease onset and 81 patients in Group S who underwent cerebral angiography between 3 and 14 days after disease onset. Patient demographic and clinical characteristics at admission, medical history, medication history, and preoperative characteristics are summarized in Table 1. Patient demographics, clinical characteristics at admission, medical history, medication history, and preoperative characteristics are summarized in Table 1. Patients in Group S were younger compared to patients in Group A (62.0 years vs. 65.0 years), had significantly lower National Institute of Health Stroke Scale (NIHSS) at admission (3 vs. 6), had significantly lower SBP at admission (140 mmHg vs. 160 mmHg), and were significantly less likely to have anterior circulation (62.9% vs. 84.9%), cardiogenic stroke (9.9% vs. 30.3%), atrial fibrillation (2.5% vs. 21.2%), or thrombolytic events (3.7% vs. 24.2%), and higher medication use of calcium channel blockers (77.6% vs. 55.6%) (Table 1).

### 4.2. Effectors of CV in SBP and DBP by Group

Results for effectors of CV in SBP and DBP during the angiography procedure are shown in Tables 2 and 3. Univariate analysis showed that age and use of calcium channel blockers (CCBs) were significantly associated with CV of SBP in Group A patients, while age and the location intracranial artery stenosis were significantly associated with CV of DBP (Table 2). Significant factors of both groups in univariate analysis were put into multivariate analysis (Table 2).

**Figure 1:**
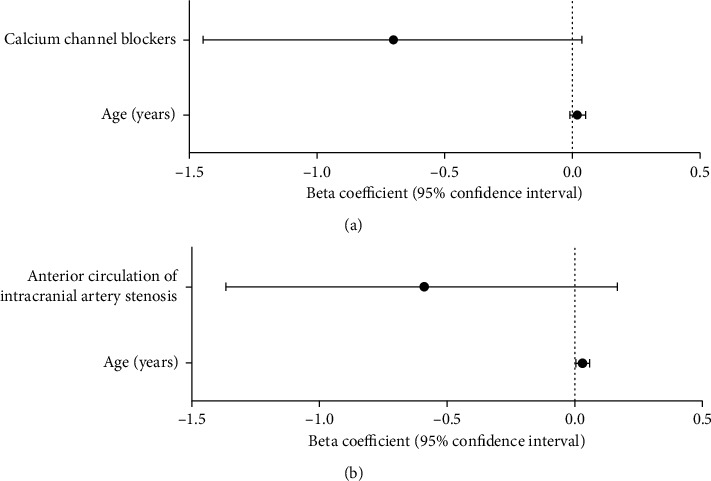
Results of multivariable analyses for coefficient of variance (CV) of systolic blood pressure (SBP) (a), coefficient of variance (CV) of diastolic blood pressure (DBP) (b) in patients receiving angiography examination within 3 days after disease onset, and the results of CV of SBP (c) in patients receiving angiography examination 3 days after (≥3 days) disease onset. Natural log transformation was applied to the CV of BP, and beta coefficients were estimated by linear regression with the backward method. CI; confidence interval; NIHSS: National Institutes of Health Stroke Scale; TOAST: Trial of ORG 10172 in acute stroke treatment.

Multivariate analysis showed that advanced age was associated with increased CV of SBP (*β* = 0.02, 95% CI = [−0.01 to 0.05]) and DBP (*β* = 0.03, 95% CI = [0.005 to 0.06]) in Group A patients. The use of CCBs was associated with lower CV of SBP (*β* = −0.70, 95% CI = [−1.45 to 0.04]) in this group, and anterior circulation of intracranial artery stenosis was associated with CV of DBP (*β* = −0.59, 95% CI = [−1.37 to 0.17]) (Figures 1(a) and 1(b)).

In group S, univariate analysis showed that use of medication for hypertension during the procedure was significantly associated with CV of SBP (Table 3).

### 4.3. Effectors of Clinical Outcomes

Patients' demographic and clinical characteristics in relation to clinical outcomes (mRS > 2) at the time of discharge are shown in Table 4. The univariate model showed that, regardless of the time interval between disease onset and angiography examination, higher NIHSS at the time of admission was significantly associated with increased risk of mRS score >2 at the time of discharge (OR = 1.37 and 1.26, 95% CI = 1.09–1.71 and 1.11–1.43, respectively) for patients taking angiography examination later.

## 5. Discussion

This prospective study investigated the effectors of BP variability during cerebral angiography and explored associations between BP variability and clinical outcomes in patients with ischemic stroke based on whether they underwent cerebral angiography within 3 days (acute) of disease onset or 3–14 days (subacute) after disease onset. In patients with intracranial artery stenosis, BP variability could be the cause of cerebral hemodynamic abnormality which potentially leads to the ischemic stroke [14]. And, BP variability during the operation can affect to the patient's prognosis [8, 15]. In order to clarify the above hypothesis, we collected and analyzed the data from 114 patients with acute and subacute ischemic stroke and intracranial artery stenosis.

Significant differences were shown between the two groups in initial characteristics at admission, BP variability, factors affecting BP and BP variability, and factors that affected patients' outcomes. However, BP variability did not affect patients' clinical outcomes at discharge, regardless of how soon after disease onset they underwent cerebral angiography.

Results of the present study also showed that male gender was associated with a lower CV of DBP compared with females among Group A patients and was associated with a lower CV of SBP among Group S patients. Advanced age also was associated with increased CV of SBP and DBP. These findings agree with those of previous studies showing that sociodemographic factors such as older age and male gender were associated with higher variability in SBP [16, 17]. In this study, the multivariate analysis showed that advanced age was associated with increased CV of SBP and DBP in Group A patients. CV of both SBP and DBP was significantly higher in patients with poor outcome compared with those with good outcome (*p* < 0.05, multivariate adjusted model). The use of CCBs was associated with lower CV of SBP in group A. Intracranial artery stenosis within anterior circulation was shown to be associated with CV of DBP. It is indicated that arterial stenosis is related to the CV of DBP and is an independent factor.

A previous systematic review of 22 studies suggested that blood pressure variability was affected by hypertension and the use of antihypertensive medications [5]. In the present study, routine use of antihypertensive medication did not significantly influence variability of SBP or DBP in Group A patients. However, in Group S patients, hypertension was significantly associated with variability of SBP, and hypertension and use of hypertension medication during the procedure were significantly associated with variability of DBP. The use of CCBs, either alone or in combination with other agents, was previously shown to effectively reduce SBP variability and prevent the incidence of stroke; positive effects of CCBs appear to be dose-dependent [18]. Interestingly, use of CCBs in the present study was significantly associated with a lower CV of SBP in Group A patients who underwent angiography within 3 days of ischemic stroke onset and with a lower CV of DBP in Group S patients whose angiography was later.

Visit-to-visit SBP variability has been shown to be a predictor of long-term all-cause and cardiovascular mortality after lacunar infarct, independent of conventional risk factors, including average BP [5]. Patients with episodic hypertension are especially noted to be at higher risk of stroke [2, 4]. In a cohort of acute ischemic stroke patients with ipsilateral intracranial cerebral artery occlusion, BP variability assessed in the acute phase was associated with poor subsequent clinical outcomes [7]. In these patients with ipsilateral intracranial cerebral artery occlusion, the effects of cerebral perfusion on BP might be more important. Since the mechanisms of autoregulation might be compromised before stroke for maintain the cerebral perfusion distally to arterial occlusion [19]. In a systematic review and meta-analysis that considered measurement techniques and definitions of BP variability along with its prognostic potential after stroke, higher systolic BP variability measured early after ischemic stroke or intracerebral hemorrhage was associated with poorer longer-term functional outcomes [14]. Those findings advised that BP variations, intracranial cerebral artery occlusion, and cerebral hemodynamic needed to be further defined and definitive measurement techniques are recommended.

BP variability after intravenous thrombolysis in acute stroke was also reported to predict poor outcomes, although it did not increase risk for intracerebral hemorrhage [15]. Moreover, about 10% of patients who needed open surgery developed preanesthesia hypertension, which was associated with 1.3% of postoperative adverse outcomes (myocardial injury/infarction or in-hospital death) [20]. However, since angiography is a mini-invasive procedure, we speculated that, unlike open surgery, BP variability during angiography may not have a significant impact on the cardiovascular or cerebrovascular systems or on clinical outcomes. In fact, results of the present study showed that BP variability during angiography was not associated with clinical outcomes at discharge. Although factors such as low baseline BP, gender, and disease severity were shown to influence BP variability during catheter angiography, this type of BP variability did not affect patients' clinical outcomes at discharge. Consistent with our results, a study of 2566 patients with acute ischemic stroke reported that BP variability was independently associated with poor functional outcome during the subacute phase (4–10 days after onset), although this was not the case during the acute phase (days 1–3 after onset) [21]. As in the present study, BP variability during the short period of catheter intervention under local anesthesia was not associated with poor clinical outcomes. Results of the present study were also compatible with those of a recent report that found no significant associations between BP variability and in-hospital outcomes in patients with acute ischemic stroke [22]. These results suggest that mild and moderate BP fluctuation during angiography does not require immediate treatment with antihypertensives, and the angiography procedure can proceed uninterrupted.

NIHSS is extensively applied in assessing clinical outcomes in stroke patients. It has also been widely used to evaluate the measurement property and accuracy of NIHSS in predicting clinical outcomes; however, it is critiqued due to its complexity and variability. Higher NIHSS scores at the time of admission were significantly associated with poor clinical outcomes for patients in both groups, regardless of how soon after disease onset cerebral angiography was performed. These data are consistent with previous findings showing that NIHSS at admission is an independent predictor of in-hospital mortality [22].

## 6. Limitations

This study has a few limitations, including that this was a single-center study with a limited number of participants of the same ethnic origins. While observing the effects of factors predicting BP variability during diagnostic cerebral angiography, we did not explore effects or cerebral dynamics associated with the particular antihypertensive drug regimens on BP variability except to note whether they occurred or not in each study group. We also did not determine the possible impact of stroke severity on BP variability and how that may also affect functional outcomes.

## 7. Conclusions

Factors associated with BP variability are significantly different between stroke patients undergoing cerebral angiography within 3 days vs. 3–14 days after disease onset. BP variability is not significantly associated with clinical outcomes at discharge. Having higher NIHSS scores at admission is associated with poor clinical outcomes regardless of the time of undergoing cerebral angiography after disease onset. Results of the present study may be useful in the clinical management of patients with acute ischemic stroke. Further studies are needed to determine the impact of BP variations on health outcomes and which factors may be more influential.

## Figures and Tables

**Table 1:** Demographic and clinical characteristics of study patients.

	Period between disease onset and angiography exam (days)		
	<3 (*n* = 33)	3–14 (*n* = 81)	*p*
Demographics			
Age, years	65.0 (57.0, 78.0)	62.0 (55.0, 72.0)	0.19^c^
Male	15 (45.45%)	36 (44.44%)	0.92^b^
Characteristics at admission			
NIHSS at admission	6.0 (4.0, 15.0)	3.0 (1.0, 6.0)	**0.0002** ^c^
SBP at admission	160.0 (143.0, 180.0)	140.0 (130.0, 160.0)	**0.004** ^c^
DBP at admission	90.0 (80.0, 91.0)	80.0 (70.0, 90.0)	**0.02** ^c^
LDL, mmol/L	3.3 ± 1.1	2.9 ± 0.8	**0.02** ^a^
Medical history			
Location of intracranial artery stenosis			0.15^b^
Posterior circulation	8 (24.24%)	31 (38.27%)	
Anterior circulation	25 (75.76%)	50 (61.73%)	
Type of TOAST			0.25^d^
Large artery atherosclerosis	25 (75.76%)	70 (86.42%)	
Small artery disease	4 (12.12%)	7 (8.64%)	
Cardiogenic stroke	4 (12.12%)	3 (3.70%)	
Others	0 (0%)	1 (1.23%)	
Anterior circulation	28 (84.85%)	51 (62.96%)	**0.02** ^b^
Hypertension	27 (81.82%)	58 (71.60%)	0.26^b^
Diabetes	10 (30.30%)	35 (43.21%)	0.20^b^
Cardiogenic stroke	10 (30.30%)	8 (9.88%)	**0.007** ^b^
Atrial fibrillation	7 (21.21%)	2 (2.47%)	**0.002** ^d^
Intravenous thrombolytics	8 (24.24%)	3 (3.70%)	**0.002** ^d^
Medication history			
Med for HTN use during operation	28 (87.50%)	67 (82.72%)	0.53^b^
Calcium channel blockers	15 (55.56%)	45 (77.59%)	**0.04** ^b^
Preoperative characteristics			
Preoperative SBP, mmHg	172.6 ± 34.7	168.8 ± 31.6	0.58^a^
Preoperative DBP, mmHg	95.3 ± 19.0	93.4 ± 15.1	0.59^a^
SBP CV, %	4.9 (2.8, 10.1)	5.9 (3.8, 10.0)	0.69^c^
DBP CV, %	8.2 (5.0, 12.8)	5.6 (3.2, 9.8)	0.07^c^

Data on continuous variables are presented as mean ± standard deviation (median (interquartile range, P25-P75)); other categorical variables are shown as frequency (%). Abbreviations: NIHSS, National Institutes of Health Stroke Scale; SBP, systolic blood pressure; DBP, diastolic blood pressure; HR: heart rate; LDL, low-density lipoprotein; TOAST, Trial of ORG 10172 in acute stroke treatment; CV, coefficient of variation. Bold values indicate statistical significance, *p* < 0.05. ^a^*T* test; ^b^chi-square test; ^c^Mann-Whitney *U* Test; ^d^Fisher's exact test.

**Table 2:** Univariate analysis for effectors^1^ of coefficient of variance (CV) of BP in patients receiving angiography examination within 3 days after disease onset.

Variables	CV of SBP	CV of DBP
	*β* (95% CI)	*β* (95% CI)
Age, years	**0.03 (0.004, 0.06)**	**0.04 (0.01, 0.06)**
Male (Ref. = female)	−0.07 (−0.83, 0.69)	−0.67 (−1.34, 0.02)
NIHSS at admission	0.03 (−0.02, 0.09)	0.05 (−0.004, 0.10)
LDL, mmol/L	0.06 (−0.37, 0.49)	0.29 (−0.10, 0.67)
Anterior circulation of intracranial artery stenosis (Ref. = posterior)	−0.24 (−1.16, 0.68)	**−0.93 (−1.73, −0.14)**
Type of TOAST (Ref. = large artery atherosclerosis)		
Small artery disease	0.36 (−0.64, 1.37)	−0.09 (−1.11, 0.93)
Cardiogenic stroke	−0.51 (−1.51, 0.49)	−0.32 (−1.34, 0.70)
Others	NA	NA
Anterior circulation (Ref. = posterior)	0.005 (−1.13, 1.14)	−0.66 (−1.73, 0.41)
Hypertension (Ref. = no)	0.48 (−0.86, 1.81)	0.42 (−0.89, 1.73)
Diabetes (Ref. = no)	−0.02 (−0.89, 0.84)	0.42 (−0.41, 1.25)
Cardiovascular (Ref. = no)	−0.36 (−1.14, 0.43)	0.41 (−0.35, 1.17)
Atrial fibrillation (Ref. = no)	0.04 (−0.79, 0.87)	0.15 (−0.66, 0.96)
Intravenous thrombolytics (Ref. = no)	−0.60 (−1.69, 0.49)	−0.17 (−1.27, 0.93)
Medication for hypertension during operation (Ref. = no)	0.78 (−1.06, 2.61)	0.12 (−1.71, 1.95)
Calcium channel blockers	**−0.87 (−1.61, 0.12)**	−0.43 (−1.24, 0.39)

Abbreviations: SBP: systolic blood pressure; DBP: diastolic blood pressure; CI; confidence interval; *β*: regression coefficient; NIHSS: National Institutes of Health Stroke Scale; LDL: low-density lipoprotein; TOAST: Trial of ORG 10172 in acute stroke treatment; NA: not available due to no event in the group. ^1^Statistics were estimated using linear regression for CV of BP. Natural log transformation was applied to the CV of BP. Bold values indicate statistical significance, *p* < 0.05.

**Table 3:** Univariate analysis for effectors^1^ of coefficient of variance (CV) of BP in patients receiving angiography examination 3 days or more after (≥3 days) disease onset.

Variables	CV of SBP	CV of DBP
	*β* (95% CI)	*β* (95% CI)
Age, years	0.001 (−0.02, 0.02)	0.0005 (−0.02, 0.02)
Male (Ref. = female)	−0.36 (−0.79, 0.08)	−0.09 (−0.56, 0.36)
NIHSS at admission	0.02 (−0.03, 0.07)	0.01 (−0.05, 0.06)
LDL, mmol/L	0.14 (−0.15, 0.43)	0.03 (−0.27, 0.34)
Anterior circulation of intracranial artery stenosis (Ref. = posterior)	0.11 (−0.35, 0.58)	0.39 (−0.08, 0.86)
Type of TOAST (Ref. = large artery atherosclerosis)		
Small artery disease	−0.32 (−1.03, 0.39)	0.25 (−0.50, 0.99)
Cardiogenic stroke	0.28 (−0.69, 1.26)	−0.02 (−1.04, 1.01)
Others	0.16 (−1.49, 1.82)	−0.02 (−1.76, 1.71)
Anterior circulation (Ref. = posterior)	−0.15 (−0.61, 0.31)	0.32 (−1.56, 0.79)
Hypertension (Ref. = no)	0.39 (−0.12, 0.92)	0.23 (−0.32, 0.78)
Diabetes (Ref = no)	0.04 (−0.41, 0.48)	−0.25 (−0.71, 0.20)
Cardiovascular (Ref. = no)	0.31 (−0.38, 1.01)	0.16 (−0.57, 0.89)
Atrial fibrillation (Ref. = no)	0.65 (−0.98, 2.28)	0.94 (−0.75, 2.63)
Intravenous thrombolytics (Ref. = no)	−0.06 (−1.69, 1.59)	0.05 (−1.65, 1.76)
Medication for hypertension during operation (Ref. = no)	**0.59 (0.12, 1.07)**	0.34 (−0.18, 0.85)
Calcium channel blockers	0.001 (−0.59, 0.60)	−0.12 (−0.74, 0.50)

Abbreviations: SBP: systolic blood pressure; DBP: diastolic blood pressure; CI; confidence interval; *β*: regression coefficient; NIHSS: National Institutes of Health Stroke Scale; LDL: low-density lipoprotein; TOAST: Trial of ORG 10172 in acute stroke treatment. ^1^Statistics were estimated using linear regression for CV of BP. Natural log transformation was applied to the CV of BP. Bold values indicate statistical significance, *p* < 0.05.

**Table 4:** Effectors^1^ of poor clinical outcomes (mRS > 2 at discharge) in stroke patients undergoing cerebral angiography.

Variables	Period between disease onset and angiography exam <3 days	Period between disease onset and angiography exam from 3 to 14 days
	OR (95% CI)	OR (95% CI)
Age, years	1.04 (0.98–1.10)	1.00 (0.97–1.04)
Male (Ref = female)	1.20 (0.29–4.82)	1.02 (0.41–2.56)
NIHSS at admission	**1.37 (1.09–1.71)**	**1.26 (1.11–1.43)**
LDL, mmol/L	0.67 (0.33–1.38)	1.74 (0.96–3.18)
Intracranial artery stenosis within anterior circulation (Ref. = posterior)	0.36 (0.06–2.15)	1.02 (0.40–2.61)
Type of TOAST (Ref. = large artery atherosclerosis)		
Small artery disease	0.79 (0.09–6.50)	0.72 (0.13–3.99)
Cardiogenic stroke	2.36 (0.21–25.91)	0.90 (0.08–10.43)
Others	NA	NA
Anterior circulation (Ref. = posterior)	0.29 (0.03–2.92)	1.51 (0.58–3.94)
Hypertension (Ref. = no)	3.40 (0.53–22.03)	1.06 (0.39–2.93)
Diabetes (Ref. = no)	2.14 (0.44–10.39)	1.11 (0.44–2.77)
Cardiovascular (Ref. = no)	4.36 (0.76–25.17)	1.92 (0.44–8.33)
Atrial fibrillation (Ref. = no)	5.99 (0.63–57.02)	1.82 (0.11–30.25)
Intravenous thrombolytics (Ref. = no)	1.31 (0.26–6.72)	0.89 (0.08–10.29)
Medication for hypertension during operation (Ref. = no)	0.39 (0.04–4.16)	1.01 (0.30–3.34)
Calcium channel blockers	0.75 (0.15–3.65)	0.88 (0.25–3.15)
SBP CV, %	1.11 (0.94–1.31)	1.02 (0.96–1.09)
DBP CV, %	1.09 (0.95–1.27)	0.95 (0.86–1.05)

Abbreviations: CV: coefficient of variance; Ref., reference group; OR, odds ratio; CI, confidence interval; HR: heart rate; LDL, low-density lipoprotein; NIHSS, National Institutes of Health Stroke Scale; SBP, systolic blood pressure; TOAST, Trial of ORG 10172 in acute stroke treatment; NA: not available due to no event in case group. ^1^All statistics were estimated by binary logistic regression. Variables with significance level <0.05 in univariable model were put into multivariable model. Bold values indicate statistical significance, *p* < 0.05.

## Data Availability

The data used to support the findings of this study are available from the corresponding author upon request and under the supervision of Shanghai Ninth People's Hospital, China.
